# Scientific abstracts and plain language summaries in psychology: A comparison based on readability indices

**DOI:** 10.1371/journal.pone.0231160

**Published:** 2020-04-02

**Authors:** Johannes Stricker, Anita Chasiotis, Martin Kerwer, Armin Günther

**Affiliations:** Leibniz Institute for Psychology Information, Trier, Germany; Johannes Gutenberg Universitat Mainz, GERMANY

## Abstract

Findings from psychological research are usually difficult to interpret for non-experts. Yet, non-experts resort to psychological findings to inform their decisions (e.g., whether to seek a psychotherapeutic treatment or not). Thus, the communication of psychological research to non-expert audiences has received increasing attention over the last years. Plain language summaries (PLS) are abstracts of peer-reviewed journal articles that aim to explain the rationale, methods, findings, and interpretation of a scientific study to non-expert audiences using non-technical language. Unlike media articles or other forms of accessible research summaries, PLS are usually written by the authors of the respective journal article, ensuring that research content is accurately reproduced. In this study, we compared the readability of PLS and corresponding scientific abstracts in a sample of 103 journal articles from two psychological peer-reviewed journals. To assess readability, we calculated four readability indices that quantify text characteristics related to reading comprehension (e.g., word difficulty, sentence length). Analyses of variance revealed that PLS were easier to read than scientific abstracts. This effect emerged in both included journals and across all readability indices. There was only little evidence that this effect differed in magnitude between the included journals. In sum, this study shows that PLS may be an effective instrument for communicating psychological research to non-expert audiences. We discuss future research avenues to increase the quality of PLS and strengthen their role in science communication.

## Introduction

Scientific journal articles in psychology are usually difficult to understand for non-experts. Yet, psychological findings influence decision-making processes in various domains, ranging from individual-level decisions (e.g., whether to seek a psychotherapeutic treatment or not) to legislation (e.g., whether to prohibit violent video games or not). Thus, there has been an increasing interest in “translating” psychological findings for non-expert audiences [1]. This endeavor has proven difficult, because, for example, media reporting frequently misrepresents scientific findings [2–4]. Hence, scientific results may be summarized incorrectly if this is done by someone other than the study authors. Moreover, scientific abstracts, written by the scientists themselves, have the disadvantage that they have become increasingly difficult to read for non-experts [5]. To bridge the communication gap between scientists and non-expert audiences, the concept of plain language summaries (PLS, also referred to as lay abstracts, non-technical summaries, impact statements or translational abstracts) has been introduced. PLS explain the rationale, methods, and findings of a scientific study using non-technical language to enable non-expert audiences to interpret scientific findings correctly. Scientific abstracts, in contrast, address an expert audience.

To date, research on PLS has largely focused on the biomedical literature [6–9]. However, psychology also constitutes a relevant field for decision-making processes, in which misconceptions are widespread [10,11] and resistant to revision [12]. Adverse effects of misunderstanding psychological findings may include, for example, ineffective teaching strategies, overreliance on witness statements in court proceedings, and misconceptions of psychological disorders [13]. Additionally, the comprehensibility of findings from social and political psychology may have consequences for socio-political processes (e.g., for predicting corrupt behavior [14]). Thus, in sum, there are many reasons why psychological researchers ought to communicate their findings in a way that is understandable for non-experts. Most psychological researchers, however, are not trained in writing for non-expert audiences. Thus, the question arises whether psychological researchers are able to simplify their writing for PLS, compared to regular scientific abstracts.

Whether psychological researchers comprehensibly summarize findings may depend on the psychological discipline. For example, a journal article in cognitive psychology may be more difficult to summarize understandably than a journal article from a more applied discipline (e.g., political psychology). Additionally, the instructions and guidance for writing PLS provided by journals may influence whether PLS are comprehensibly written or not.

As a proxy for text comprehensibility (i.e., how easily information recipients will be able to extract information from a text), readability indices (i.e., scores based on surface-level text characteristics) are frequently used. For example, the Cochrane Collaboration, an independent international organization that produces high-quality and accessible systematic reviews to inform evidence-based health decision making, recommends the Simple Measure of Gobbledygook (SMOG [15]) to assess the comprehensibility of PLS [16]. The SMOG is a readability index which is based on the proportion of words with three or more syllables. Beyond the context of PLS, the SMOG has been used to assess the readability of health information materials [17], educational materials [18], and psychometric instruments [19]. Besides the SMOG, various other readability indices exist, which quantify text characteristics related to reading comprehension. Among the most widely used readability indices are the Flesch Reading Ease Score (FRES [20]), the Flesch-Kincaid Readability Score (FKRS [21]), and the New Dale–Chall Readability Formula (NCDRF [22]). Even though readability indices may only approximate text comprehensibility, there is considerably evidence for their validity [23,24], including positive correlations with text comprehension judgments in adult readers [25].

Previous research on differences in readability between PLS and scientific abstracts has focused on Cochrane systematic reviews and has produced mixed findings. Cochrane systematic reviews synthesize and appraise evidence to inform health decisions using systematic and reproducible methods. These reviews either focus on the harms and benefits of interventions in healthcare (intervention reviews) or on the accuracy of diagnostic tests for diagnosing a particular disease (diagnostic test accuracy reviews). A recent investigation found lower SMOG scores (indicating better reliability) for a sample of 156 PLS of Cochrane systematic reviews compared to the corresponding scientific abstracts [6]. However, in a different study, there was no statistically significant difference between PLS and scientific abstracts in SMOG scores in a sample of 84 Cochrane diagnostic test accuracy reviews (systematic reviews summarizing evidence about test accuracy) [7]. Regarding actual information extraction, one study found that university students more easily extract information from PLS compared to scientific abstracts of Cochrane systematic reviews, also reporting a better reading experience and higher user-friendliness [8]. Yet, a different study on university students’ information extraction from Cochrane systematic reviews found neither a statistically significant difference between PLS and scientific abstracts for Cochrane systematic reviews with clear findings, nor for Cochrane systematic reviews with unclear findings [9].

In psychology, PLS are supposed to enable non-experts to understand and draw conclusions from research findings. Thus, they should exhibit a higher readability than traditional scientific abstracts. The aim of the present study was to compare the readability of scientific abstracts and PLS in psychology based on reproducible readability indices.

## Method

### Identification of PLS and corresponding scientific abstracts

To identify psychological peer-reviewed journals that provide PLS, we conducted an explorative web-based search in June 2019 and contacted experts from different psychological disciplines. Various journals include a brief statement (i.e., one to three sentences) regarding the public significance of an article (e.g., Journal of Abnormal Psychology, Journal of Applied Sports Psychology). Other journals (e.g., Psychological Methods) provide non-technical summaries explicitly directed at other researchers rather than non-expert audiences. We have identified only two psychological peer-reviewed journals with PLS, whose length and content correspond to that of the corresponding scientific abstracts and which are aimed at non-experts: Archives of Scientific Psychology and the Journal of Social and Political Psychology. Archives of Scientific Psychology is the American Psychological Association’s (APA) open-access journal publishing articles from all psychological disciplines. The Journal of Social and Political Psychology is an online open-access journal (without author fees) publishing articles with different methodical and theoretical perspectives at the intersection of social and political psychology. The editors of Archives of Scientific Psychology ask authors to provide a PLS for the educated public describing the study and its relevance for societal or psychological problems. Additionally, the editors offer assistance in writing PLS, if necessary. For the Journal of Social and Political Psychology, a PLS is optional, but strongly encouraged. The editors of the Journal of Social and Political Psychology ask authors to provide a PLS for lay audiences to increase the visibility of their work beyond the respective discipline and beyond academia more generally. To structure PLS, the Journal of Social and Political Psychology provides mandatory sub-headings: 1. Background, 2. Why was this study done, 3. What did the researchers do and find, 4. What do these findings mean.

### Retrieval of PLS and corresponding scientific abstracts

Overall, we retrieved 103 PLS and corresponding scientific abstracts in February 2020 (67 from Archives of Scientific Psychology, 36 from the Journal of Social and Political Psychology). To retrieve the scientific abstracts and PLS from Archives of Scientific Psychology, we used PsycINFO. We retrieved the scientific abstracts and PLS from the Journal of Social and Political Psychology from the journal’s database on the PsychOpen GOLD platform operated by the Leibniz Institute for Psychology Information (ZPID). Prior to calculating readability indices, we removed the standardized sub-headings (i.e., 1. Background, 2. Why was this study done, 3. What did the researchers do and find, 4. What do these findings mean?) from the PLS of articles in the Journal of Social and Political Psychology as not discarding them would have biased resulting readability indices.

### Readability indices

In accordance with the Cochrane Collaboration’s recommendation [16], we used the SMOG to compare the readability of scientific abstracts and PLS. To test, whether obtained effect estimates depended on the selection of this specific readability index, we additionally repeated all analyses with three other frequently used readability indices: The FRES, the FKRS, and the NDCRF. The readability indices were calculated with the quanteda package [26] in the R statistical environment [27]. The SMOG is based on the number of words with three syllables or more relative to the number of sentences in a text. Lower SMOG scores indicate better readability. We calculated the SMOG with the following formula: $\text{SMOG}=1.043\;\text{x}\;\sqrt{n_{\text{wsy}>=3}}\;\text{x}\;\frac{30}{n_{\text{st}}}+3.1291$, where *n*_wsy> = 3_ is the number of words with three syllables or more and *n*_st_ is the number of sentences. The FRES is based on average sentence length and the number of syllables per word. Higher FRES scores indicate better reliability. We calculated the FRES with the following formula: $\text{FRES}=206.835-\left(1.015\times \text{ASL}\right)-(84.6\times \frac{n_{sy}}{n_{w}})$ where ASL is the average sentence length (number of words/number of sentences), *n*_sy_ is the number of syllables, and *n*_w_ is the number of words. The FKRS is also based on average sentence length and number of syllables per word. Lower FKRS scores indicate better readability. We calculated the FKRS as $0.39\times \text{ASL}+11.8\times \frac{n_{sy}}{n_{w}}-15.59$. Finally, the NDCRF is based on the number "difficult" words not matching the Dale-Chall list of 3,000 "familiar" words relative to the overall number of words. The Dale-Chall list contains all words that 80% of fourth-grade students were familiar with in a validation study [22]. Higher NCDRF scores indicate better reliability. We calculated the NCDRF using the following formula: $\text{NDCRF}=64-\left(0.95\times 100\times \frac{n_{wd}}{n_{w}}\right)$ − (0.69×ASL), where *n*_wd_ is the number of "difficult" words not matching the Dale-Chall list of 3,000 "familiar" words [22].

### Statistical analyses

We conducted a 2 (abstract type) × 2 (journal) mixed analysis of variance (ANOVA) with abstract type (scientific abstract, PLS) as within-factor, journal (Archives of Scientific Psychology, Journal of Social and Political Psychology) as between-factor and the SMOG as dependent variable. A significant main effect of abstract type indicates that scientific abstracts and PLS differ in readability. A significant main effect of journal indicates that there is a difference between the two journals regarding the readability of PLS and scientific abstracts. A significant interaction of abstract type and journal indicates that differences in readability between the articles’ scientific abstracts and PLS are more accentuated in one journal compared to the other journal. As follow up analyses, we conducted one-way ANOVAs with abstract type (scientific abstract, PLS) as within-factor and the SMOG as dependent variable for each journal separately. To test the robustness of our analyses, we repeated all analyses with the FRES, the FKRS, and the NDCRF as dependent variables. Our data and scripts are available via the PsychArchives repository:, http://doi.org/10.23668/psycharchives.2768, http://dx.doi.org/10.23668/psycharchives.2723. We deleted all scientific abstracts and PLS for Archives of Scientific Psychology from the data set prior to uploading. This was due to PsycINFO's copyright policy. The scientific abstracts and PLSs can be found via the DOIs we provided.

## Results

### Preliminary analyses

The average text length was 183.04 words (*SD* = 44.00) for scientific abstracts and 277.96 words (*SD* = 153.81) for PLS. Table 1 displays the means and standard deviations for all readability indices by abstract type and journal. Table 2 displays the bivariate correlations among the four readability indices for scientific abstracts and PLS. There was a medium correlation between the SMOG of a scientific abstract and the SMOG of the corresponding PLS (*r* = .33, *p* < .001). The four readability indices were highly correlated (|*r*| ≥ .68, *p*s < .001 for scientific abstracts and |*r*| ≥ .76, *p*s < .001 for PLS).

**Table 1:** Means and standard deviations for all readability indices by abstract type and journal.

Readability index	Scientific abstracts						Plain language summaries					
	ASP		JSPP		Overall		ASP		JSPP		Overall	
	*M*	*SD*	*M*	*SD*	*M*	*SD*	*M*	*SD*	*M*	*SD*	*M*	*SD*
SMOG	17.82	1.96	18.43	2.33	18.03	2.11	17.03	2.18	17.08	1.91	17.04	2.08
FRES	17.73	13.08	11.01	13.49	15.38	13.55	23.50	12.64	23.74	12.62	23.58	12.57
FKRS	17.36	2.78	18.11	2.69	17.62	2.76	16.47	3.19	16.27	2.34	16.40	2.91
NDCRF	3.34	8.53	3.05	6.70	3.24	7.90	9.08	8.09	11.17	4.87	9.81	7.17

ASP = Archives of Scientific Psychology. JSPP = Journal of Social and Political Psychology. SMOG = Simple Measure of Gobbledygook. FRES = Flesch Reading Ease Score. FKRS = Flesch-Kincaid Readability Score. NDCRF = New Dale–Chall Readability Formula.

**Table 2:** Bivariate correlation matrix of the readability indices.

Readability index (abstract type)	1	2	3	4	5	6	7	8
1. SMOG (scientific abstract)	-							-
2. FRES (scientific abstract)	-.86***	-						
3. FKRS (scientific abstract)	.93***	-.85***	-					
4. NDCRF (scientific abstract)	-.72***	.68***	-.78***	-				
5. SMOG (PLS)	.33**	-.37***	.25*	-.18	-			
6. FRES (PLS)	-.31**	.44***	-.27**	.20*	-.91***	-		
7. FKRS (PLS)	.22*	-.26**	.20*	-.14	.93***	-.85***	-	
8. NDCRF (PLS)	-.14	.18	-.13	.29**	-.77***	.76***	-.82***	-

SMOG = Simple Measure of Gobbledygook. FRES = Flesch Reading Ease Score. FKRS = Flesch-Kincaid Readability Score. NDCRF = New Dale–Chall Readability Formula. PLS = Plain language summary.

*p < .05.

**p < .01.

***p < .001.

### Differences in readability

A 2 (abstract type) × 2 (journal) mixed ANOVA with abstract type (scientific abstract, PLS) as within-factor, journal (Archives of Scientific Psychology, Journal of Social and Political Psychology) as between-factor, and the SMOG as dependent variable revealed a significant main effect of abstract type, indicating that PLS are easier to read than scientific abstracts, *F*(1, 101) = 18.47, *p* < .001, *η*_*p*_^2^ = .16. The main effect of journal did not reach statistical significance, indicating that there was no significant difference between the two journals in the readability of scientific abstracts and PLS, *F*(1, 101) = 0.88, *p* = .349, *η*_*p*_^2^ = .01. Moreover, the interaction effect of abstract type and journal did not reach statistical significance, *F*(1, 101) = 1.26, *p* = .264, *η*_*p*_^2^ = .01. This finding indicates that the difference in readability between an article’s scientific abstract and PLS did not differ significantly between the two journals. Follow-up one-way ANOVAs with abstract type (scientific abstract, PLS) as within-factor confirmed the significant effect of abstract type for each journal separately, *F*(1, 66) = 5.80, *p* = .019, *η*_*p*_^2^ = .08 for Archives of Scientific Psychology and *F*(1, 35) = 20.86, *p* < .001, *η*_*p*_^2^ = .37 for the Journal of Social and Political Psychology.

### Robustness analyses

In a set of 2 (abstract type) × 2 (journal) mixed ANOVAs with abstract type as within-factor and journal as between factor, we found significant main effects of abstract type for the three additional readability indices, *F*(1, 101) = 44.30, *p* < .001, *η*_*p*_^2^ = .31 for the FRES, *F*(1, 101) = 13.63, *p* < .001, *η*_*p*_^2^ = .12 for the FKRS, and *F*(1, 101) = 55.58, *p* < .001, *η*_*p*_^2^ = .36 for the NDCRF. For Archives of Scientific Psychology, follow-up one-way ANOVAs with abstract type as within-factor confirmed a better readability of PLS compared to scientific abstracts for the FRES (*F*(1, 66) = 10.16, *p* = .002, *η*_*p*_^2^ = .13) and the NDCRF (*F*(1, 66) = 20.97, *p* < .001, *η*_*p*_^2^ = .24), but not for the FKRS (*F*(1, 66) = 3.13, *p* = .082, *η*_*p*_^2^ = .05). For the Journal of Social and Political Psychology, follow-up one-way ANOVAs with abstract type as within-factor confirmed a better readability of PLS compared to scientific abstracts for the FRES (*F*(1, 35) = 53.89, *p* < .001, *η*_*p*_^2^ = .61, the FKRS (*F*(1, 35) = 24.71, *p* < .001, *η*_*p*_^2^ = .41), and the NDCRF (*F*(1, 35) = 67.48, *p* < .001, *η*_*p*_^2^ = .66 for the NDCRF) separately. In sum, these findings suggest that the readability of PLS is higher than the readability of scientific abstracts across all included readability indices and in in both included journals. The main effect of journal did not reach significance for any of the readability indices, *F*(1, 101) = 2.01, *p* = .159, *η*_*p*_^2^ = .02 for the FRES, *F*(1, 101) = 0.39, *p* = .534, *η*_*p*_^2^ = .00 for the FKRS, and *F*(1, 101) = 0.52, *p* = .474, *η*_*p*_^2^ = .01 for the NCDRF. The interaction effect of abstract type and journal reached statistical significance for the FRES (*F*(1, 101) = 6.28, *p* = .014, *η*_*p*_^2^ = .06), but not for the FKRS (*F*(1, 101) = 1.65, *p* = .202, *η*_*p*_^2^ = .02) or the NCDRF (*F*(1, 101) = 1.64, *p* = .203, *η*_*p*_^2^ = .02). Thus, there is only little evidence that the magnitude of the difference in readability between an article’s scientific abstract and the corresponding PLS differs between the two journals.

## Discussion

### The potential future role of PLS in psychological science communication

This study showed that PLS in two psychological peer-reviewed journals are easier to read than scientific abstracts. This effect was evident for each of the two journals and replicated across all readability indices. Thus, psychological researchers seem to be able to communicate their findings in a language more accessible for non-experts compared to the language used in scientific abstracts. Against the background of the increasing relevance of science communication [28], this finding seems encouraging. One advantage of PLS over other types of science communication is that they are automatically linked to the relevant journal articles. Thus, PLS can prevent misinterpretations of research findings that can easily arise when non-experts interpret scientific journal articles. The relevance of PLS for science communication may further increase, because, in the context of open access publishing, a growing proportion of psychological articles are accessible free of charge to broad audiences online. In writing PLS, however, it may also be challenging for researchers not to over-simplify complex issues and to communicate scientific uncertainty accurately [29–31]. Scientific uncertainty may also discourage researchers from communicating their findings to the public [32]. In this regard, PLS are practicable in that information can be prepared for the public without a researcher having to communicate proactively via (social) media. Providing a PLS may also prevent misinterpretations of research findings in the media, a scenario often feared by researchers [33].

Although we could only include two psychological journals in this study, the finding that PLS are easier to read than scientific abstracts might generalize beyond these journals across different disciplines of psychology. This assumption is supported by the great variety in content and methods of articles published in both included journals. Thus, editors of journals from all psychological disciplines might consider encouraging authors to submit a PLS together with their journal articles.

### Limitations and future research directions

This study has several limitations. First, we did not assess actual information extraction by non-experts. Thus, the text characteristics quantified in this study might be unrelated to non-experts’ comprehension of PLS and scientific abstracts. However, assessing the SMOG is consistent with the Cochrane Collaboration’s recommendation [16] and quantified text characteristics are related to text comprehension in adult readers [25]. Future studies are needed to replicate our findings based on non-experts’ information extraction from scientific abstracts and PLS. Second, there is no threshold regarding absolute readability scores that could indicate acceptable readability for non-expert audiences. Thus, both scientific abstracts and PLS may be difficult to understand for non-experts. Initially, readability indices were developed to assess the number of years in formal education (grade level) required to comprehend a text. However, this interpretation of readability indices has been thoroughly criticized [34]. Particularly in brief texts, the assignment of a specific grade level is unreliable [35]. Future research is needed to establish thresholds indicating acceptable readability for non-expert audiences. There is some evidence that readers of scientific content designed for non-experts are generally highly educated [36]. Thus, it will be important to clarify who exactly is the target audience of PLS. Additionally, the SMOG scores of the psychological journal articles included in this study were higher than previously reported SMOG scores of Cochrane systematic reviews [6,7], indicating lower readability. Future research is needed to test whether the readability of PLS differs systematically between different scientific fields (e.g., medicine and psychology) or research methods (e.g., primary studies and systematic reviews). Third, there was only little evidence that the difference in readability between scientific abstracts and the corresponding PLS depended on the journal an article was published in. However, given the pattern of results obtained in our robustness analyses, we cannot rule out that this effect might have become (consistently) significant in a larger sample. Fourth, this study provided no information regarding the instructions that authors need to compose comprehensible PLS. To date, it is unclear whether differences in author instructions might influence the readability of PLS. This question could not be resolved in this study because the two included journals differed not only regarding author instructions, but also regarding their content area and authors. Additionally, it is unclear to which extent assistance by the journal editors was requested and how this assistance improved PLS readability. For the Journal of Social and Political Psychology, there might have been a selection effect: As a PLS was not strictly required in this journal, authors with difficulties in writing PLS might have omitted it. Future studies in which components of author instructions are experimentally manipulated might reveal which information is essential for researchers to write comprehensible PLS. These experimental studies could additionally test which elements of PLS (e.g., subheadings) increase text comprehension. Fifth, this study did not assess which author characteristics are related to improved readability of PLS compared to scientific abstracts. For example, it might be easier for researchers from applied research fields to write comprehensible PLS compared to researchers in basic research. Similarly, experiences in public engagement or specific writing training might increase the readability of PLS. Future research could develop and evaluate training programs on science communication (e.g., in undergraduate and graduate courses [37]) that increase the willingness and ability to write PLS.

Against the background of the increasing importance of science communication, this study showed that, in two psychological peer-reviewed journals, author-written PLS are easier to read than scientific abstracts. We hope that this finding aids in strengthening the role of study authors as “public communicators” [38] of their research.
